# Evaluation of the Antitumor Efficacy of Human Papillomavirus Type 16 E7-Affitoxin in C57BL/6JNifdc Mice Bearing TC‑1 Tumors

**DOI:** 10.1021/acs.molpharmaceut.5c01013

**Published:** 2025-10-06

**Authors:** Hua Zhu, Zhihui Zhang, Jingwei Ye, Hongshuai Yang, Jingjie Lin, Xinlei Cao, Lifang Zhang, Yubing Chen, Pengfei Jiang

**Affiliations:** 1 Department of Microbiology and Immunology, School of Basic Medical Sciences, 26453 Wenzhou Medical University, Wenzhou, Zhejiang 325035, P. R. China; 2 Department of Gynecology, 89657 The First Affiliated Hospital of Wenzhou Medical University, Wenzhou 325035, China; 3 Department of Clinical Laboratory, Yantai Affiliated Hospital of Binzhou Medical University, Yantai 264100, China

**Keywords:** HPV16, affitoxin, antitumor targeted therapy, C57BL/6JNifdc mice, TC-1 tumor

## Abstract

Human papillomavirus (HPV) associated cancers pose a significant global health threat, with HPV 16 being the most common causative type. Current treatments often lack specificity and cause severe side effects. In a previous study, we developed an HPV16 E7 targeted therapeutic agent, Z_HPV16E7_ affitoxin384, which effectively inhibited tumor growth in immunodeficient nude mice bearing HPV16-positive cervical tumors. In this study, we further evaluated the antitumor efficacy of Z_HPV16E7_ affitoxin384 in immunocompetent C57BL/6JNifdc mice bearing TC-1 tumors. Z_HPV16E7_ affitoxin384 had demonstrated specific binding to HPV16 E7 in TC-1 cells, significantly inhibiting their proliferation and promoting apoptosis both in vitro and in vivo. In C57BL/6JNifdc mice, Z_HPV16E7_ affitoxin384 effectively suppressed TC-1 tumor growth, with a therapeutic effect comparable to that of cisplatin. Acute toxicity tests indicated dose-dependent toxicity, but no significant organ damage or effects on liver/kidney function or blood parameters were observed at tested doses. This study provides robust evidence supporting Z_HPV16E7_ affitoxin384 as a promising targeted therapy for HPV16-induced cancers, highlighting its potential for future clinical applications.

## Graphical abstract

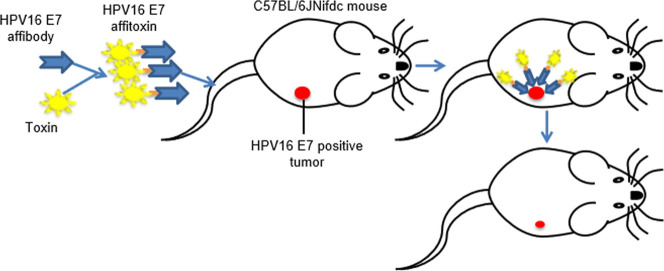

## 1 Introduction

Human papillomavirus (HPV) is predominantly a sexually transmitted infection responsible for at least seven types of cancers in both men and women, including cervical, oropharyngeal, head and neck, vaginal, anal, penile, and vulvar cancers.^1,2^ Globally, over 5% of human cancers and 95% of cervical cancer cases are attributed to infections caused by 14 high-risk HPV types,^2,3^ among which HPV 16 alone accounts for 60% of cervical cancer cases.^4,5^ Despite the availability of prophylactic vaccines, insufficient global vaccine coverage results in more than 600,000 new cases of cervical cancer and over 34,000 deaths in 2020.^6^ Current treatments for HPV-related precancerous lesions and cancers, such as surgery, radiotherapy, and chemotherapy, are often associated with significant side effects.^7^ Therefore, the development of targeted therapeutic agents for HPV-induced diseases remains a critical unmet need.

The HPV E7 protein plays a pivotal role in the pathogenesis of HPV-related cancers by binding to and inactivating the retinoblastoma (Rb) protein, thereby disrupting cell cycle regulation and promoting malignant transformation.^8^ In addition to its interaction with Rb, E7 engages with numerous other cellular proteins, with further study needed for its oncogenic potential. Thus, HPV E7 is considered as an ideal target for the development of therapeutic agents against HPV-related diseases.^8^

Affibodies, a class of engineered affinity proteins with only 58 amino acid residues, have been used as an alternative to antibodies for biotechnological and medical applications because of their several advantages over conventional antibodies.^9^ As such, affibodies represent a set of promising agents for next-generation targeted cancer therapies.^9^ To enhance their therapeutic efficacy, affibodies are frequently conjugated with cytotoxic molecules. *Pseudomonas aeruginosa* exotoxin A (PEA) is a bacterial toxin that enters host cells via receptor-mediated endocytosis and is processed by furin proteases, the resulting fragments of which catalyze the ADP-ribosylation of eukaryotic elongation factor 2, leading to the inhibition of protein synthesis and subsequent cell death. The modified PEA (PE38KDEL) has demonstrated significant therapeutic potential in preclinical models and clinical trials.^10^

In a previous study, we developed an affibody molecule, Z_HPV16E7_384, which specifically binds to HPV16 E7.^11^ By conjugating Z_HPV16E7_384 with PE38KDEL, we created Z_HPV16E7_ affitoxin384, a targeted therapeutic agent that effectively inhibited tumor growth in immunodeficient nude mice bearing HPV16-positive cervical tumors.^12^ However, as nude mice lack a functional immune system and inadequately replicate the tumor microenvironment,^13,14^ we extended our investigations to evaluate the therapeutic efficacy of Z_HPV16E7_ affitoxin384 in immunocompetent C57BL/6JNifdc mice. Through comprehensive in vitro and in vivo studies, we aim to provide robust theoretical and experimental evidence supporting Z_HPV16E7_ affitoxin384 as a potential targeted therapy for HPV16 E7-positive tumors.

## 2 Materials and Methods

### 2.1 Animals, Cells, and Vectors

Female C57BL/6JNifdc mice, 6 to 8 weeks old, were purchased from Beijing Vital River Laboratory Animal Technology Co., Ltd. and kept at the animal facility of Wenzhou Medical University, China. All animal experiments were approved by the Institutional Animal Care and Use Committee of Wenzhou Medical University (Ethics Approval Number: wydw2021–0445). The TC-1 cell line, generated by transformation of C57BL/6 mouse lung epithelial cells with HPV16 E7, was a kind gift from Dr. Xuemei Xu (Peking Union Medical College, Beijing, China). The B16 cell line was used as the HPV16 E7 negative control cell line. The recombinant plasmids pET21a (+)/Z_HPV16E7_ affitoxin384 and pET21a (+)/Z_wt_ affitoxin, respectively, encoding Z_HPV16E7_ affitoxin384 and Z_wt_ affitoxin, were constructed as reported previously.^12^

### 2.2 Reagents

The reagents used, including Cell Counting Kit-8 (Dojindo, Japan), RPMI-1640 (Gibco, USA), fetal bovine serum (Gibco, USA), trypsin-EDTA (Gibco, USA), isopropyl-beta-D-thiogalactopyranoside (IPTG) (Sigma-Aldrich, Saint Louis, USA), Ni-NTA agarose (Qiagen Inc., Valencia, CA), and Annexin V-FITC/PI Apoptosis Kit (Hangzhou Lianke Biotechnology Co., Ltd., China), were purchased from commercial sources. The anti-HPV16 E7 mouse monoclonal antibody (Santa Cruz Biotechnology (Shanghai) Co., Ltd., China) and anti-His tag mouse monoclonal antibody, goat antirabbit IgG (H + L) conjugated with HRP and goat antimouse IgG (H + L) conjugated with HRP, and goat antimouse antibody conjugated with FITC and goat antirabbit antibody conjugated with Cy3 (Hangzhou Lianke Biotechnology Co., Ltd., China) were purchased from commercial sources.

### 2.3 Expression and Purification of Z_HPV16E7_ Affitoxin384 and Z_wt_ Affitoxin

The expression of Z_HPV16E7_ affitoxin384 and Z_wt_ affitoxin in *E. coli* BL21 (DE3) was induced by 1 mM IPTG and verified by SDS-PAGE and Western blot analysis. Then, the proteins were purified by a Ni-NTA Sepharose column.

### 2.4 Western Blot Analysis

Western blotting was performed to analyze the indicated proteins. All samples were run on 12% SDS-polyacrylamide gel and transferred onto polyvinylidene difluoride membranes (Millipore, USA). Membranes were blocked with 5% skim milk in TBST (1 × TBS + 0.1% Tween-20) for 2 h, incubated with the indicated primary antibodies, and then incubated with the HRP-conjugated secondary antibody. The protein bands were visualized using 0.005% (w/v) 4-chloro-1-naphthol and a 0.015% (v/v) hydrogen peroxidase color development substrate.

### 2.5 Indirect Immunofluorescence Assay

To evaluate the in vitro targeting ability of Z_HPV16E7_ affitoxin384, TC-1 and B16 cells were seeded in confocal dishes. After 24 h of incubation, the cells were treated with 2 μM Z_HPV16E7_ affitoxin384 or Z_wt_ affitoxin for 6 h. Then, an indirect immunofluorescence assay (IFA) was performed as described in a previous study.^15^

### 2.6 In Vitro Efficacy of Z_HPV16E7_ Affitoxin384

To evaluate the efficacy of Z_HPV16E7_ affitoxin384, a cell viability assay was performed with Cell Counting Kit-8 (CCK-8) (Dojindo) as described in a previous study.^12^ TC-1 cells were treated with Z_HPV16E7_ affitoxin384 at different concentrations (0, 0.125, 0.25, 0.5, 1, 2, and 4 μM) to analyze its half maximal inhibitory concentration (IC_50_). IC_50_ values were calculated using GraphPad Prism software (GraphPad Software, Inc.). TC-1 and B16 cells were seeded into a 96-well plate at 4000 cells per well, followed by treatment with Z_HPV16E7_ affitoxin384 at the concentration of IC_50_. Cell viability was determined after incubation for 12, 24, 36, and 48 h. Cells treated with the same concentration of Z_wt_ affitoxin were used as negative controls.

The effect of Z_HPV16E7_ affitoxin384 on the proliferation of target cells was detected by colony formation and 5-ethynyl-2′-deoxyuridine (EdU) assays. For the colony formation assay, TC-1 and B16 cells were separately seeded into 6-well plates at a density of 1000 cells per well, followed by treatment with 2 μM Z_HPV16E7_ affitoxin384 for 14 days. The colonies were stained and calculated as described in a previous study.^15^ For the EdU assay, TC-1 and B16 cells were separately seeded into 24-well plates at a density of 1 × 10^5^ cells per well. After 24 h, the cells were exposed to 2 μM Z_HPV16E7_ affitoxin384 in a complete medium for 48 h. Cell proliferation was detected by the incorporation of EdU as described in a previous study.^15^ TC-1 and B16 cells treated with Z_wt_ affitoxin or PBS were used as negative controls.

### 2.7 Flow Cytometry

TC-1 and B16 cells were separately seeded into six-well plates with 1 × 10^5^ cells per well. The cells were treated with Z_HPV16E7_ affitoxin384 at the concentration of IC_50_ for 24 h. Apoptosis was analyzed with an Annexin V-FITC/PI Apoptosis Kit (Hangzhou Lianke Biotechnology Co., Ltd., China) as described in a previous study.^16^

### 2.8 Evaluation of Z_HPV16E7_ Affitoxin384 Acute Toxicity

C57BL/6JNifdc female mice (*n* = 5 per group) were administered with the indicated doses (200, 400, 600, 800, and 1000 nmol/kg) of Z_HPV16E7_ affitoxin384 by intravenous injection into the tail vein. Any reported death cases or moribund conditions that occurred within the 2-week post injection period were taken into consideration. All experiments were performed in triplicate.

Blood samples were collected at day 14 postinjection in EDTA tubes for complete blood count and in plain tubes for liver and kidney function panel tests. Then, blood samples in EDTA tubes were analyzed using a Mindray BC-5380 Hematology Analyzer (Shenzhen Mindray Bio-Medical Electronics Co., Ltd., Shenzhen, China), and serums generated from blood samples in plain tubes were analyzed using a Beckman AU680 Chemistry Analyzer (Beckman Coulter, Brea, CA, USA).

### 2.9 In Vivo Antitumor Efficacy of Z_HPV16E7_ Affitoxin384

The therapeutic efficacy of Z_HPV16E7_ affitoxin384 was studied using TC-1 tumor-bearing mice. C57BL/6JNifdc mice were randomly divided into 4 groups (*n* = 4 per group). The tumors were initiated by subcutaneous injection of 5 × 10^6^ cells, which were suspended in 0.1 mL of PBS, into the right forelimb of each mouse. Tumor dimensions were measured periodically using calipers, and their volumes were calculated using the following formula: volume = length × width^2^ × 0.52. When the tumor size reached 50–100 mm^3^ in diameter, mice were treated with 0.2 mL of Z_HPV16E7_ affitoxin384 (400 nmol/kg), Z_wt_ affitoxin (400 nmol/kg), cisplatin (400 nmol/kg), or PBS. The indicated agents were injected every 2 days for five time points via tail vein. The therapeutic efficacies and systemic toxicities of affitoxin proteins were evaluated based on daily measurements of tumor volume and body weight. Tumors from mice in the aforementioned four groups were separated and weighed after all treatments and observations were finished.

### 2.10 Statistical Analysis

Data are presented as mean ± SD. Statistical analysis of the significance between groups was conducted using Student's *t* test, and *p* < 0.05 was considered to be statistically significant. All calculations were performed with the software SPSS25.0.

## 3 Results

### 3.1 Successful Preparation of Z_HPV16E7_ Affitoxin384

The pET21a (+)/Z_HPV16E7_ affitoxin384 and pET21a (+)/Z_wt_ affitoxin plasmids were transformed into prokaryotic expression strain *E. coli* BL21. Following induction with IPTG for 6 h, the protein expressions of Z_HPV16E7_ affitoxin384 and Z_wt_ affitoxin were analyzed by sodium dodecyl sulfate polyacrylamide gel electrophoresis (SDS-PAGE). Distinct protein bands corresponding to Z_HPV16E7_ affitoxin384 and Z_wt_ affitoxin were observed at approximately 45 kDa (Figure 1A), consistent with their predicted molecular weights. Both plasmids encode a C-terminal His tag, enabling purification of the proteins by affinity chromatography. The purity and identity of the purified proteins were confirmed by SDS-PAGE and Western blot analysis (Figure 1B,C).

**Figure 1:**
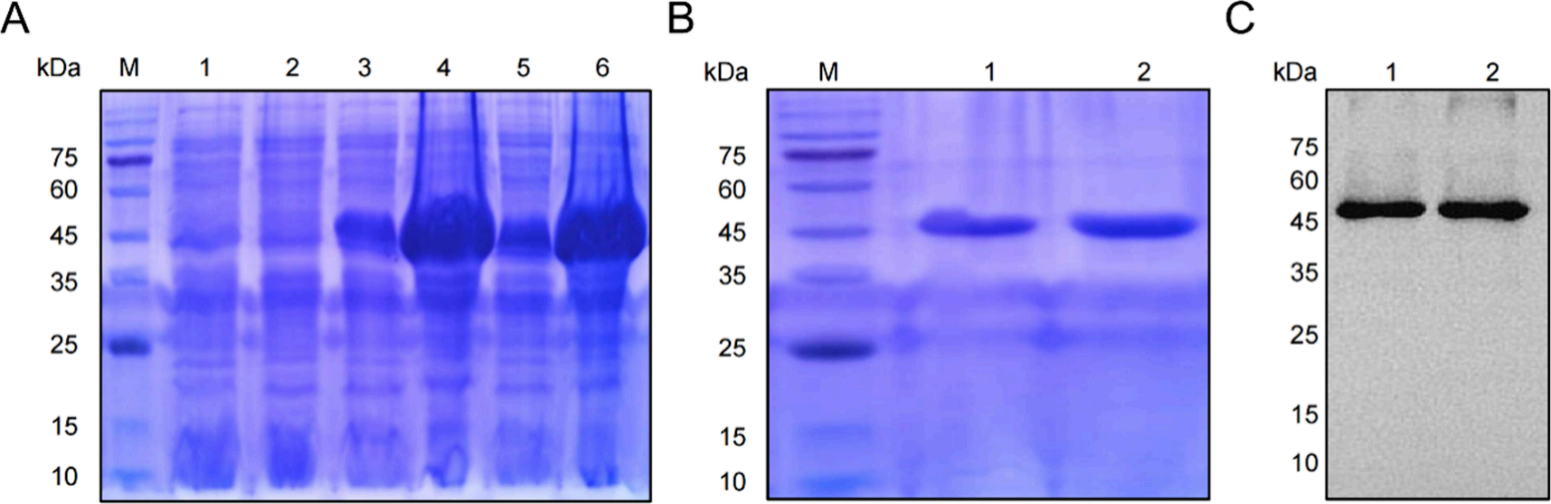
Expression and purification of Z_HPV16E7_ affitoxin384. (A) Coomassie blue-stained SDS-PAGE gel of the recombinant proteins. M, protein marker; 1, *E. coli* BL21 (DE3); 2, pET21a (+)/*E. coli* BL21 (DE3); 3, pET21a (+)/Z_wt_ affitoxin/*E. coli* BL21 (DE3) without IPTG induction; 4, pET21a (+)/Z_wt_ affitoxin/*E. coli* BL21 (DE3) with IPTG induction; 5, pET21a (+)/Z_HPV16E7_ affitoxin384/*E. coli* BL21 (DE3) without IPTG induction; 6, pET21a (+)/Z_HPV16E7_ affitoxin384/*E. coli* BL21 (DE3) with IPTG induction. (B) SDS-PAGE analysis of purified recombinant proteins. M, protein marker; 1, purified Z_wt_ affitoxin; 2, purified Z_HPV16E7_ affitoxin384. (C) Western blot analysis of purified recombinant proteins. 1, purified Z_wt_ affitoxin; 2, purified Z_HPV16E7_ affitoxin384.

### 3.2 Z_HPV16E7_ Affitoxin384 Specifically Targets HPV16 E7 Protein in TC-1 Cells

TC-1 cells, derived from primary C57BL/6JNifdc mouse lung epithelial cells cotransformed with HPV16 E6/E7 and activated ras oncogene,^17^ were used as target cells for Z_HPV16E7_ affitoxin384. B16 melanoma cells, which do not express HPV16 E7, served as a negative control. HPV16 E7 expressed in TC-1 cells was confirmed by RT-PCR, Western blot, and IFA (Figure 2A–C). These results demonstrated that TC-1 and B16 cells could be used for further studies.

**Figure 2:**
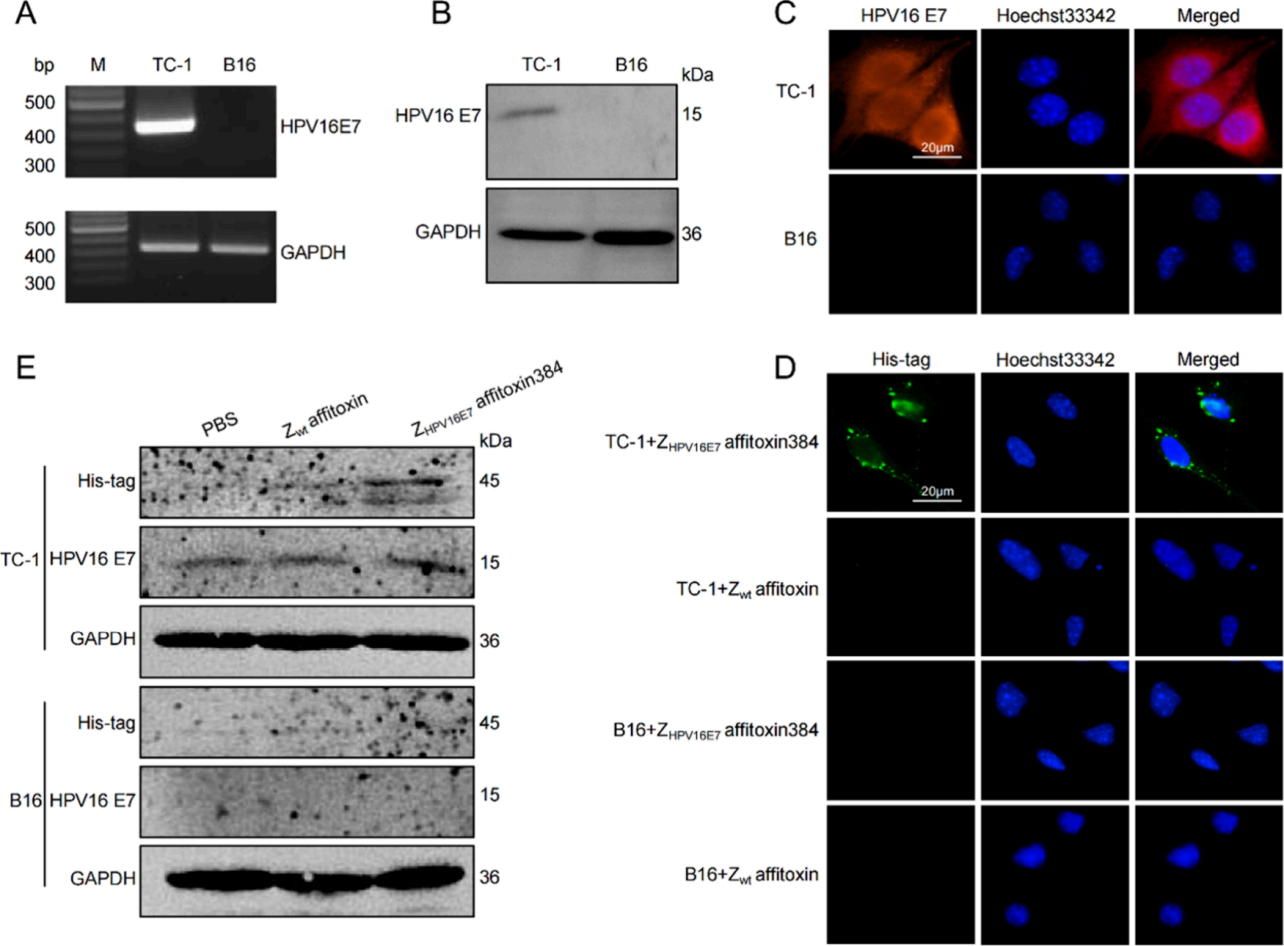
Specific binding of Z_HPV16E7_ affitoxin384 to HPV16 E7 proteins. (A) RT-PCR analysis of HPV16 E7 gene expression in TC-1 cells. B16 cells were used as a negative control. (B) Western blot analysis of HPV16 E7 protein expression in TC-1 cells. B16 cells were used as a negative control. (C) IFA analysis of the HPV16 E7 protein expression in TC-1 cells. B16 cells were used as a negative control. (D) IFA analysis of the binding specificity of Z_HPV16E7_ affitoxin384 to HPV16 E7. TC-1 cells incubated with Z_HPV16E7_ affitoxin384 were used as the test group, and TC-1 cells incubated with Z_wt_ affitoxin and B16 cells incubated with either Z_HPV16E7_ affitoxin384 or Z_wt_ affitoxin served as controls. The anti-His tag antibody was used as the primary antibody. (E) Western blot analysis of the binding specificity of Z_HPV16E7_ affitoxin384 to HPV16 E7. TC-1 cells and B16 cells were incubated with PBS, Z_wt_ affitoxin, or Z_HPV16E7_ affitoxin384, followed by Western blot analysis using the primary antibodies against anti-His tag, HPV16 E7, and GPADH.

To assess the binding specificity of Z_HPV16E7_ affitoxin384 to HPV16 E7, TC-1 and B16 cells were incubated with Z_HPV16E7_ affitoxin384 or Z_wt_ affitoxin for 6 h, followed by IFA using an anti-His tag monoclonal antibody as the primary antibody. Distinct green fluorescence signals, indicative of Z_HPV16E7_ affitoxin384 binding, were observed in the cytoplasm of TC-1 cells treated with Z_HPV16E7_ affitoxin384. In contrast, no fluorescence was detected in TC-1 cells treated with Z_wt_ affitoxin or in B16 cells treated with either Z_HPV16E7_ affitoxin384 or Z_wt_ affitoxin (Figure 2D). These results indicate that Z_HPV16E7_ affitoxin384 can specifically target and bind to the HPV16 E7 protein in TC-1 cells.

To further validate the binding affinity and specificity, Western blot was performed. TC-1 and B16 cells were incubated with Z_HPV16E7_ affitoxin384 or Z_wt_ affitoxin for 6 h, followed by cell lysis and protein extraction. Using the anti-His tag monoclonal antibody, a distinct band at 45 kDa, corresponding to Z_HPV16E7_ affitoxin384, was detected in TC-1 cells treated with Z_HPV16E7_ affitoxin384. No such band was observed in the control groups (Figure 2E). These findings further confirm the specific binding of Z_HPV16E7_ affitoxin384 to HPV16 E7 in TC-1 cells.

### 3.3 Z_HPV16E7_ Affitoxin384 Specifically Inhibits the Proliferation of TC-1 Cells

To quantify the inhibitory effect on target cells, the IC_50_ of Z_HPV16E7_ affitoxin384 on TC-1 cells was determined. TC-1 cells were treated with Z_HPV16E7_ affitoxin384 at concentrations ranging from 0.1 to 10 μM for 72 h, followed by cell viability analysis. As shown in Figure 3A, the IC_50_ value of Z_HPV16E7_ affitoxin384 for TC-1 cells was calculated to be 3.889 μM. Then, to evaluate the effect of Z_HPV16E7_ affitoxin384 on TC-1 cells, TC-1 and B16 cells were incubated with Z_HPV16E7_ affitoxin384 or Z_wt_ affitoxin at a concentration of 3.889 μM for 0, 12, 24, 36, and 48 h. Cell viability was detected using the CCK-8 assay. As shown in Figure 3B, Z_HPV16E7_ affitoxin384 significantly reduced the viability of TC-1 cells, while Z_wt_ affitoxin had no significant effect. Statistical analysis revealed significant differences between the two groups at each time point. In contrast, the treatment of B16 cells with either Z_HPV16E7_ affitoxin384 or Z_wt_ affitoxin showed no significant impact on cell viability (Figure 3C). These results demonstrate that Z_HPV16E7_ affitoxin384 selectively inhibits the viability of HPV16 E7-positive TC-1 cells.

**Figure 3:**
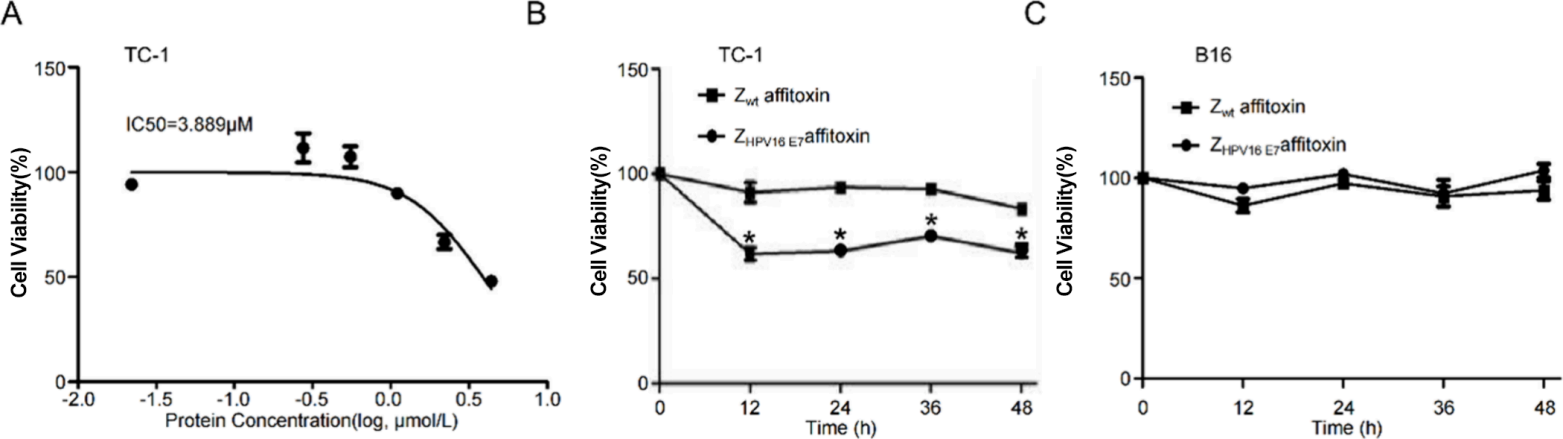
Z_HPV16E7_ affitoxin384 significantly inhibits the viability of target cells. (A) Determination of the IC_50_ value of Z_HPV16E7_ affitoxin384 in TC-1 cells. Data are representative of three independent experiments. (B) Viabilities of TC-1 cells treated with indicated concentrations of Z_wt_ affitoxin or Z_HPV16E7_ affitoxin384 for 48 h were analyzed by the CCK-8 assay. (C) Viabilities of B16 cells treated with indicated concentrations of Z_wt_ affitoxin or Z_HPV16E7_ affitoxin384 for 48 h were analyzed by the CCK-8 assay. All experiments were performed in triplicate, and data are expressed as means ± SD (*n* = 3). *p < 0.05.

### 3.4 Z_HPV16E7_ Affitoxin384 Significantly Inhibits the Proliferation of Target Cells

To evaluate the effect of Z_HPV16E7_ affitoxin384 on target cell proliferation, a colony formation assay was performed. TC-1 and B16 cells were incubated with Z_HPV16E7_ affitoxin384, Z_wt_ affitoxin, or PBS used as the controls. As shown in Figure 4A,B, TC-1 cells treated with Z_HPV16E7_ affitoxin384 formed significantly fewer and smaller cell colonies compared to those treated with Z_wt_ affitoxin or PBS. In contrast, there was no significant difference in colony formation in B16 cells treated with Z_HPV16E7_ affitoxin384, Z_wt_ affitoxin, or PBS.

**Figure 4:**
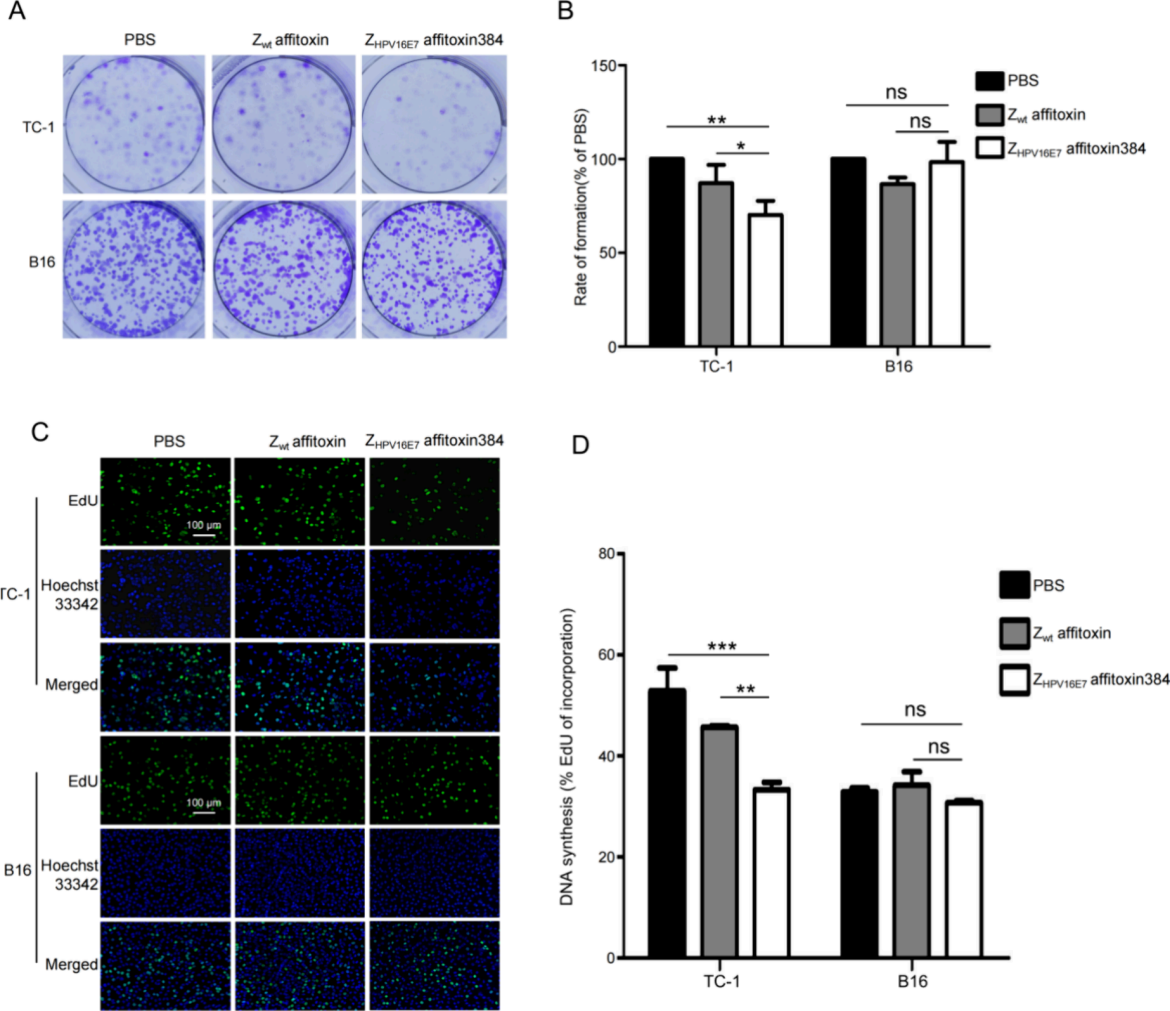
Z_HPV16E7_ affitoxin384 significantly inhibits the proliferation of target cells. (A) Colony formation assay of TC-1 and B16 cells treated with PBS, Z_wt_ affitoxin, and Z_HPV16E7_ affitoxin384 was performed to assess the antiproliferative effect of Z_HPV16E7_ affitoxin384 on HPV16 E7 positive TC-1 cells. (B) Quantification of colony numbers from (A). (C) EdU proliferation assay of Z_HPV16E7_ affitoxin384 on cell proliferation. Cell proliferation was determined by the incorporation of EdU (green). Cell nuclei were counterstained with Hoechst 33342 (Blue). Scale bar = 100 μm. (D) Quantification of the percentage of EdU-positive cells from (C). Data are representative of three independent experiments. All experiments were performed in triplicate, and data are expressed as means ± SD (*n* = 3), *p < 0.05, **p < 0.01, ***p < 0.001, ns: no significant difference.

The inhibitory effect of Z_HPV16E7_ affitoxin384 on target cell proliferation was further confirmed by using the EdU assay. As shown in Figure 4C,D, the percentage of EdU-positive TC-1 cells was significantly reduced following treatment with Z_HPV16E7_ affitoxin384 compared to that of the controls. However, no such effect was observed in B16 cells. These results collectively demonstrate that Z_HPV16E7_ affitoxin384 selectively inhibits the proliferation of HPV16 E7-positive TC-1 cells.

### 3.5 Z_HPV16E7_ Affitoxin384 Promotes Apoptosis of TC-1 Cells

To evaluate the effect of Z_HPV16E7_ affitoxin384 on apoptosis, flow cytometry analysis was performed. As shown in Figure 5A,B, treatment with Z_HPV16E7_ affitoxin384 significantly increased both the early apoptosis rate and the late apoptosis rate in TC-1 cells compared to treatment with Z_wt_ affitoxin. In contrast, no significant difference in the apoptosis rate was observed in B16 cells treated with Z_HPV16E7_ affitoxin384 or Z_wt_ affitoxin (Figure 5C,D).

**Figure 5:**
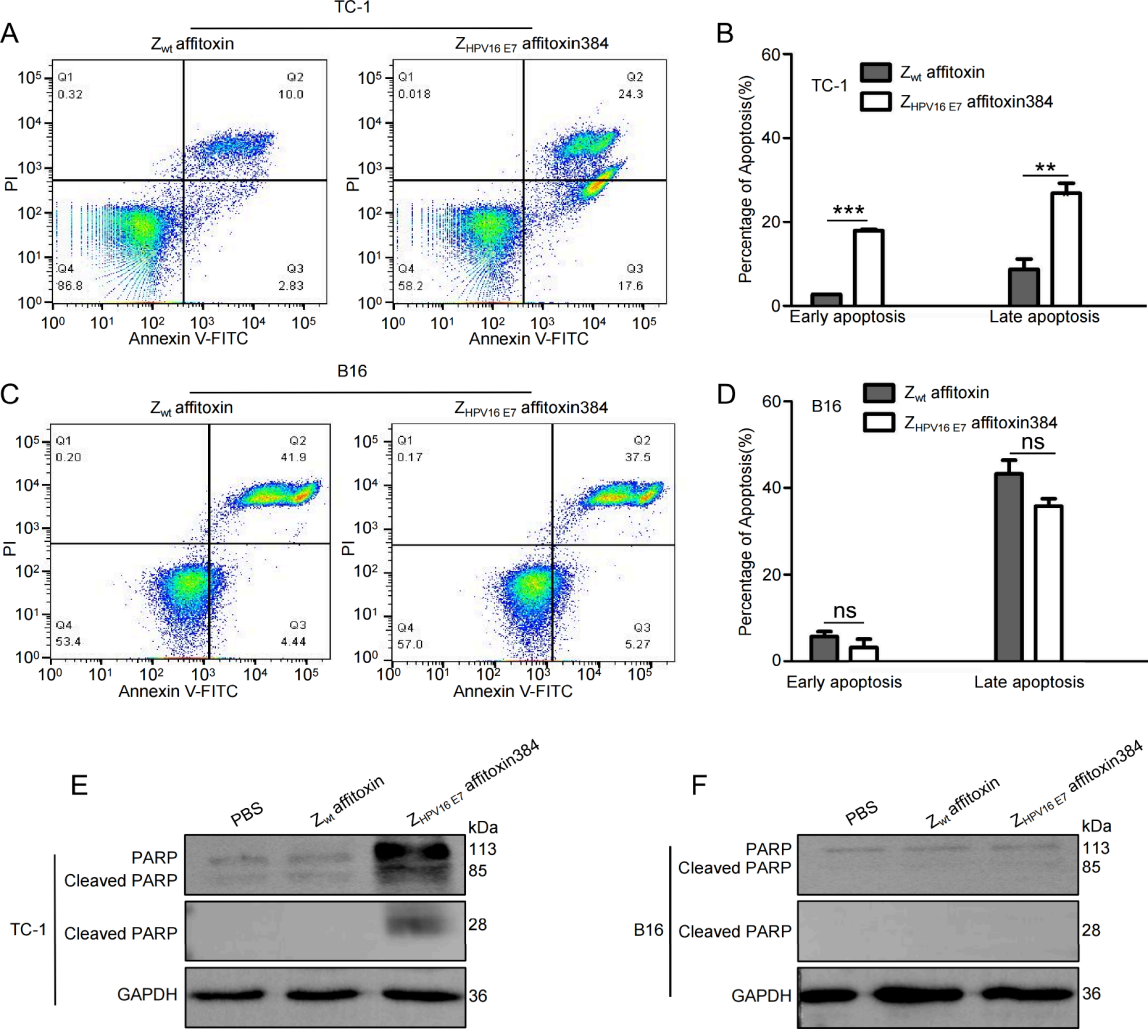
Z_HPV16E7_ affitoxin384 promotes apoptosis of TC-1 cells (A) Flow cytometry analysis of apoptosis in TC-1 cells treated with Z_HPV16E7_ affitoxin384. Cells treated with Z_wt_ affitoxin served as controls. (B) Quantification of apoptosis rates in TC-1 cells from (A). Data are expressed as mean ± SD (*n* = 3), **p < 0.01, ***p < 0.001. (C) Flow cytometry analysis of apoptosis in B16 cells treated with Z_HPV16E7_ affitoxin384. Cells treated with Z_wt_ affitoxin served as controls. (D) Quantification of apoptosis rates in B16 cells from (C). Data are expressed as mean ± SD (*n* = 3), ns: no significant difference. (E,F) Western blot analysis of PARP cleavage in TC-1 and B16 cells treated with PBS, Z_wt_ affitoxin, or Z_HPV16E7_ affitoxin384. Full-length PARP (113 kDa), cleaved PARP (85 kDa and 28 kDa), and GAPDH were detected using rabbit-derived and mouse-derived monoclonal antibodies.

To further confirm the proapoptotic effect of Z_HPV16E7_ affitoxin384, Western blot analysis was performed to detect the cleavage of poly­(ADP-ribose) polymerase (PARP), a key substrate of caspase-3 and a hallmark of apoptosis. Full-length PARP (113 kDa) is cleaved into 85 kDa and 28 kDa fragments during apoptosis. As shown in Figure 5E,F, TC-1 cells treated with Z_HPV16E7_ affitoxin384 exhibited clear PARP cleavage bands at 85 kDa and 28 kDa, whereas few cleavage was observed in control groups. These results further demonstrate that Z_HPV16E7_ affitoxin384 promotes apoptosis in HPV16 E7-positive TC-1 cells.

### 3.6 Z_HPV16E7_ Affitoxin384 Significantly Suppresses Tumor Growth in TC-1 Tumor-Bearing Mice

To investigate the effect of Z_HPV16E7_ affitoxin384 on the growth of TC-1 tumors in C57BL/6JNifdc mice, we administered Z_HPV16E7_ affitoxin384 and other controls on days 0, 2, 4, 6, and 8 to mice with tumors at volumes of 50–100 mm^3^. As shown in Figure 6A, TC-1 tumor growth in mice treated with Z_HPV16E7_ affitoxin384 and cisplatin was significantly slower compared to that treated with Z_wt_ affitoxin or PBS, with differences becoming apparent from day 6. By day 10, tumor volumes in the Z_HPV16E7_ affitoxin384 and cisplatin groups were significantly smaller than those in the Z_wt_ affitoxin and PBS groups. In contrast, for B16 tumors, a significant difference in tumor volume was observed only between the cisplatin group and the other groups (Figure 6D). As depicted in Figure 6B, the smallest tumor volume in TC-1 tumors was observed in the cisplatin group, followed by that in the Z_HPV16E7_ affitoxin384 group. Similarly, in B16 tumors, the cisplatin group also showed the smallest tumor volume (Figure 6E). Furthermore, in TC-1 tumors, tumor weights in the Z_HPV16E7_ affitoxin384 and cisplatin groups were significantly lower than those in the Z_wt_ affitoxin and PBS groups (Figure 6C). In B16 tumors, only the cisplatin group showed a significant reduction in tumor weight compared with the other groups (Figure 6F). These results suggest that Z_HPV16E7_ affitoxin384 significantly inhibits the growth of TC-1 tumors in C57BL/6JNifdc mice.

**Figure 6:**
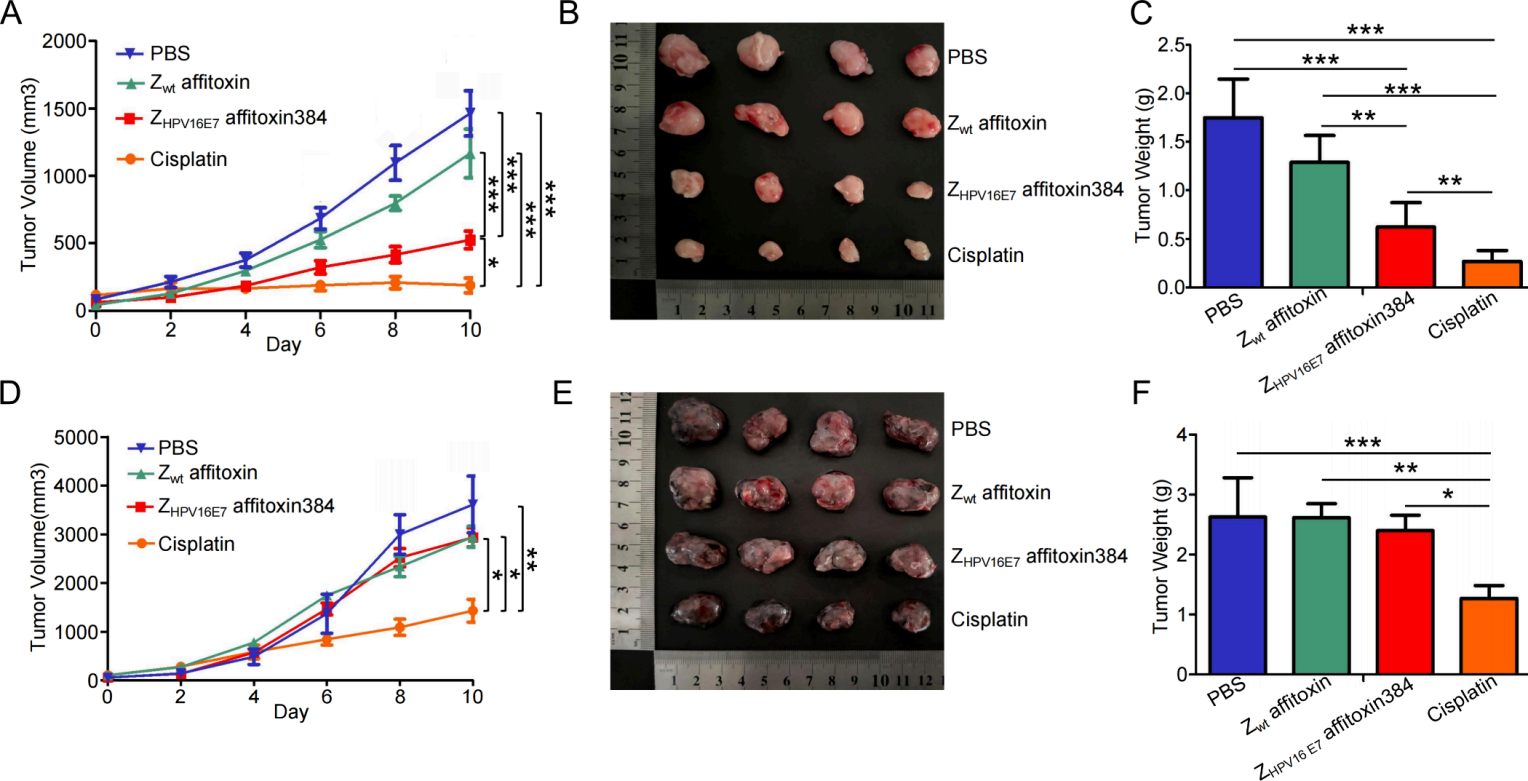
In vivo antitumor efficacy of Z_HPV16E7_ affitoxin384. Therapeutic efficacy of Z_HPV16 E7_ affitoxin384 was studied using TC-1 and B16 tumor-bearing mice. Tumor-bearing mice were prepared in advance, followed by injection of PBS, Z_wt_ affitoxin, Z_HPV16E7_ affitoxin384, or cisplatin every 2 days for 5 times via tail vein. The therapeutic efficacy was evaluated based on daily measurements of tumor volume and body weight. (A) Tumor volume growth curves for TC-1 tumors. (B) Tumors from mice in (A) were separated. (C) All tumors from (B) were weighed and compared. (D) Tumor volume growth curves for B16 tumors. (E) Tumors from mice in (D) were separated. (F) All tumors from (E) were weighed and compared. Data are given as mean ± SD (*n* = 4), *p < 0.05, **p < 0.01, ***p < 0.001.

To evaluate the acute toxicity of Z_HPV16E7_ affitoxin384 and determine the optimal therapeutic dose for tumor-bearing mice, C57BL/6JNifdc female mice were divided into five test groups and a PBS control group, with 5 mice per group. Mice were observed continuously for 14 days following protein injection. As shown in Table 1, in the 200 and 400 nmol/kg dose groups and the control PBS group, no mouse deaths were observed. However, mice in the three higher dose groups (600, 800, and 1000 nmol/kg) died within 30 min postinjection, while surviving mice exhibited signs of lethargy and reduced activity. These results indicate the dose-dependent toxicity of Z_HPV16E7_ affitoxin384, highlighting the need for careful dose optimization in therapeutic applications.

**Table 1:** Toxicity of Z_HPV16E7_ Affitoxin384 to C57BL/6JNifdc Mice

dose of Z_HPV16E7_ affitoxin384 (nmol/kg)	PBS	200	400	600	800	1000
mortality (%)	0	0	0	20	60	60

To assess the potential organ toxicity of Z_HPV16E7_ affitoxin384, all mice were deceased and dissected after 14 days postinjection, and their organs were collected to calculate the visceral indexes. As shown in Table 2, except for a slight increase in the thymus index in the 1000 nmol/kg dose group, no significant difference was observed in visceral indexes between the other dose groups and the PBS control group. These results suggest that Z_HPV16E7_ affitoxin384 did not cause significant organ damage in mice at the tested doses.

**Table 2:** Visceral Indexes of C57BL/6JNifdc Mice Injected with Different Doses of Z_HPV16E7_ Affitoxin384^a^

	dose of Z_HPV16E7_ affitoxin384 (nmol/kg)					
lists	PBS	200	400	600	800	1000
heart	0.50 ± 0.09	0.46 ± 0.03	0.44 ± 0.04	0.53 ± 0.05	0.44 ± 0.09	0.55 ± 0.11
liver	4.95 ± 0.49	4.31 ± 0.37	4.41 ± 0.54	4.72 ± 0.56	5.45 ± 0.59	5.10 ± 0.77
spleen	0.37 ± 0.05	0.40 ± 0.09	0.37 ± 0.06	0.47 ± 0.08	0.40 ± 0.05	0.42 ± 0.14
lungs	0.63 ± 0.05	0.62 ± 0.10	0.59 ± 0.05	0.65 ± 0.09	0.62 ± 0.05	0.62 ± 0.06
kidney	1.15 ± 0.05	1.14 ± 0.04	1.09 ± 0.02	1.21 ± 0.15	1.29 ± 0.13	1.31 ± 0.20
thymus	0.24 ± 0.04	0.26 ± 0.02	0.29 ± 0.04	0.27 ± 0.04	0.25 ± 0.07	0.32 ± 0.07

a All experiments were performed in triplicate, and data are expressed as means ± SD (*n* = 4).

### 3.7 Effects of Z_HPV16E7_ Affitoxin384 on Liver and Kidney Function in Mice

To evaluate the potential impact of Z_HPV16E7_ affitoxin384 on liver and kidney function, blood samples were collected from mice in variant dose groups (200, 400, and 600 nmol/kg) and the PBS control group via retro-orbital bleeding after 14 days postinjection. Liver and kidney function markers were analyzed, and the results are summarized in Table 3. No significant difference was observed between the dose groups and the control group, indicating that Z_HPV16E7_ affitoxin384 does not significantly affect liver or kidney function in mice at the tested doses.

**Table 3:** Liver and Kidney Function Panel Performed for Normal C57BL/6JNifdc Mice Injected with Different Doses of Z_HPV16E7_ Affitoxin384^a^

	dose of Z_HPV16E7_ affitoxin384 (nmol/kg)			
lists	PBS	200	400	600
alanine aminotransferase (U/L)	42.45 ± 6.47	36.28 ± 3.16	33.80 ± 5.94	37.53 ± 4.24
aspartate aminotransferase (U/L)	160.53 ± 44.40	150.73 ± 7.56	143.78 ± 40.74	194.05 ± 33.19
urea nitrogen (mmol/L)	13.03 ± 2.26	9.12 ± 0.80	14.16 ± 7.93	10.79 ± 3.32
creatinine (μmol/L)	19.20 ± 7.45	11.55 ± 0.26	11.65 ± 1.09	15.75 ± 1.87
albumin (g/L)	36.68 ± 1.27	37.78 ± 1.96	36.67 ± 1.80	36.50 ± 2.86
total protein (g/L)	63.41 ± 4.28	68.50 ± 2.12	64.88 ± 2.94	63.77 ± 5.84

a All experiments were performed in triplicate, and data are expressed as means ± SD (*n* = 4).

The above blood samples were also used to assess the potential impact of Z_HPV16E7_ affitoxin384 on blood routine parameters. The results are summarized in Table 4. No significant difference was observed between the dose groups and the control group, indicating that Z_HPV16E7_ affitoxin384 did not significantly affect blood routine parameters in mice at the tested doses.

**Table 4:** Complete Blood Count Performed for C57BL/6JNifdc Mice Injected with Different Doses of Z_HPV16E7_ Affitoxin384^a^

	dose of Z_HPV16E7_ affitoxin384 (nmol/kg)			
lists	PBS	200	400	600
white blood cell count (×10^9^ cells/L)	3.75 ± 1.24	3.85 ± 1.05	3.45 ± 1.23	4.78 ± 1.25
lymphocyte count (×10^9^ cells/L)	3.10 ± 1.06	3.05 ± 1.01	2.73 ± 0.90	3.73 ± 0.92
neutrophil count (×10^9^ cells/L)	0.40 ± 0.14	0.48 ± 0.28	0.38 ± 0.21	0.65 ± 0.24
red blood cell count (×10^12^ cells/L)	8.26 ± 0.92	9.32 ± 0.65	9.00 ± 0.49	8.98 ± 0.53
hemoglobin (g/L)	144.75 ± 18.5	158.50 ± 13.70	153.75 ± 4.65	150.00 ± 6.06
hematocrit (%)	42.05 ± 5.10	45.18 ± 262	4330 ± 205	4580 ± 431
mean corpuscular volume (fL)	48.35 ± 1.03	48.50 ± 0.84	48.13 ± 0.75	50.98 ± 2.57
mean corpuscular hemoglobin (pg)	16.63 ± 1.05	16.98 ± 0.41	17.10 ± 0.49	16.70 ± 0.49
mean corpuscular hemoglobin concentration (g/L)	343.75 ± 24.54	350.00 ± 12.25	355.25 ± 9.78	328.75 ± 23.87
platelet count (×10^9^/L)	843.00 ± 162.37	502.25 ± 344.20	639.25 ± 436.25	562.25 ± 104.46

a All experiments were performed in triplicate, and data are expressed as means ± SD (*n* = 4).

## 4 Discussion

High-risk HPV infection is causally linked to approximately 5% of cancers worldwide.^3^ Among high-risk HPV types, HPV 16 is the most carcinogenic and predominant in HPV-related cancers.^3^ Early diagnosis and HPV vaccines are crucial for preventing these diseases. However, low vaccination rates and limitations in HPV-positive cancer screening have hindered prevention efforts, particularly in underdeveloped regions.^18,19^ Current cancer treatments, such as surgery, radiotherapy, and chemotherapy, often lack specificity, leading to significant side effects. In recent years, a variety of innovative approaches have emerged in the field of tumor treatment including target therapy, gene therapy, oncolytic virus therapy, and CAR-T cell therapy, each with its unique advantages and limitations.^20–24^ In these approaches, targeted therapies offer a more precise approach by blocking specific cancer-related signaling pathways or proteins, inducing apoptosis, stimulating the immune system, or delivering chemotherapeutic agents directly to cancer cells, thereby minimizing off-target effects.^25^ Among these, affibodies have emerged as a promising class of agents for targeted payload delivery.^9^ Recent studies have demonstrated the potential of affibodies in targeting HPV oncoproteins for the treatment of HPV-positive tumors.^26,27^

The HPV E7 protein has been well established as a key player in the pathogenesis of HPV-related cancers.^8^ Given its essential role in tumorigenesis, HPV E7 has emerged as an ideal target for therapeutic intervention.^8^ In a previous study, we developed an affinity agent, Z_HPV16E7_ affitoxin384, which specifically and efficiently inhibited HPV16 E7-positive tumors through targeted therapy in cervical cancer-bearing nude mice.^12^ However, given the limitations of immunodeficient models, they inadequately replicate the tumor microenvironment.^13,14^ In contrast, C57BL/6JNifdc mice, with their intact immune system, provide a more physiologically relevant model for studying the tumor microenvironment and evaluating antitumor drug efficacy.^28^

Therefore, in order to evaluate the efficacy of Z_HPV16E7_ affitoxin384 from nude mice model in the previous study^12^ and provide a reference for future clinical trials, C57BL/6JNifdc mice was utilized in this study to assess the therapeutic potential of Z_HPV16E7_ affitoxin384 to HPV16 E7-positive tumors. However, in immune-competent mice, xenograft tumor models could not be established using human cervical cancer SiHa or CaSki cells expressing the HPV16 E7 protein as target cells for Z_HPV16E7_ affitoxin384 due to rejection responses. The TC-1 cell line, derived from mouse lung epithelial cells stably expressing HPV16 E7,^17^ was chosen for its ability to mimic key characteristics of HPV-associated tumors in an immune-competent system.^29,30^

Affinity and specificity are critical determinants for the applicability of targeted diagnostics and therapeutics. Z_HPV16E7_ affitoxin384 exhibits high and specific affinity to HPV16 E7-positive cancer cells.^12^ Consistent with the previous study, the affinity and specificity of Z_HPV16E7_ affitoxin384 to HPV16 E7 in TC-1 cells were confirmed in this study. Z_HPV16E7_ affitoxin384 selectively bound to and inhibited the viability and proliferation of HPV16 E7-positive TC-1 cells but showed negligible effects on HPV16 E7-negative B16 cells both in vitro and in vivo, demonstrating its high specificity and therapeutic potential. The PE38KDEL domain in Z_HPV16E7_ affitoxin384 could induce apoptosis in target cells by inhibiting protein synthesis.^10^ Flow cytometry analysis confirmed that Z_HPV16E7_ affitoxin384 significantly induced early and late apoptosis in TC-1 cells, with no notable effects on B16 cells, further validating its targeted cytotoxicity. PARP is a DNA repair enzyme, cleavage of which by caspases is a prominent characteristic of apoptosis.^31^ In this study, cleaved PARP was significantly induced in TC-1 cells with Z_HPV16E7_ affitoxin384 treatment. These results demonstrated that Z_HPV16E7_ affitoxin384 effectively targets and binds to HPV16 E7, selectively suppressing the proliferation of HPV16 E7-positive cells. Thus, these findings also highlight the potential of a precise therapeutic agent for HPV16-associated cancers.

Additionally, we conducted a comprehensive toxicity assessment of Z_HPV16E7_ affitoxin384 to provide valuable data for its clinical trials. Injection with higher doses of Z_HPV16E7_ affitoxin384 caused mouse deaths to varying degrees, with the mortality rate rising as the concentration of Z_HPV16E7_ affitoxin384 increased. However, no significant toxic effects were observed in visceral indexes, liver and kidney function, or blood parameters on day 14 postinjection. Combined with the results in our previous study, nude mice injected with the Z_HPV16E7_ affitoxin384 had elevated AST on day 1 and significantly higher blood urea nitrogen on days 3 and 7, whereas these liver and kidney function indicators were statistically similar to the control group on day 14.^12^ These results illustrated that only high doses of Z_HPV16E7_ affitoxin384 administered to mice could cause liver and kidney damage in a short period, thus resulting in the deaths of mice. It is worth noting that Z_HPV16E7_ affitoxin384 was administered via the tail vein in this study. As reported, local administration is an effective method to increase the concentration of agents in tumor tissues and minimize undesirable systematic distribution, thereby improving safety.^32^ Therefore, local administration will be an alternative method to decrease the dose of Z_HPV16E7_ affitoxin384 and reduce the mortality rate in our future study. Although cisplatin showed greater efficacy at equivalent doses, the reduced systemic toxicity and targeted specificity of Z_HPV16E7_ affitoxin384 position it as a compelling candidate for combination therapies or as a safer alternative to HPV-related cancer treatment.

However, repeated administration of Z_HPV16E7_ affitoxin384 may induce humoral immune responses, potentially limiting its therapeutic efficacy. While our study underscores its excellent therapeutic effects and low toxicity in immunocompetent mice, the development of a low-immunogenicity version of Z_HPV16E7_ affitoxin384 is essential for its clinical application. This also opens new avenues for research to optimize its therapeutic potential in HPV-associated cancers.

## 5 Conclusions

In conclusion, the present study investigated the antitumor efficacy of Z_HPV16E7_ affitoxin384 in C57BL/6JNifdc mice bearing TC-1 tumors. Our findings demonstrated that Z_HPV16E7_ affitoxin384, an HPV16 E7 targeted therapeutic agent, successfully inhibited HPV16 E7-positive tumor growth in immunocompetent mice. This study further supports the potential of Z_HPV16E7_ affitoxin384 as a promising therapeutic agent for HPV16 induced cervical cancer.

## ABBREVIATIONS

HPV: human papillomavirus
Rb: retinoblastoma
PEA: *Pseudomonas aeruginosa* exotoxin A
PE38KDEL: modified PEA
SDS-PAGE: sodium dodecyl sulfate polyacrylamide gel electrophoresis
IFA: indirect immunofluorescence assay
IC50: half maximal inhibitory concentration
PARP: poly­(ADP-ribose) polymerase
CCK-8: Cell Counting Kit-8
IPTG: isopropyl-beta-D-thiogalactopyranoside
EdU: 5-ethynyl-2′-deoxyuridine
Z_HPV16E7_ affitoxin384: protein encoded by pET21a (+)/Z_HPV16E7_ affitoxin384 vector
Z_wt_ affitoxin: protein encoded by pET21a (+)/Z_wt_ affitoxin vector
PI: propidium iodide
h: hours
